# Molecular Optical Simulation Environment (MOSE): A Platform for the Simulation of Light Propagation in Turbid Media

**DOI:** 10.1371/journal.pone.0061304

**Published:** 2013-04-08

**Authors:** Shenghan Ren, Xueli Chen, Hailong Wang, Xiaochao Qu, Ge Wang, Jimin Liang, Jie Tian

**Affiliations:** 1 School of Life Sciences and Technology, Xidian University, Xi’an, Shaanxi, China; 2 Institute of Automation, Chinese Academy of Sciences, Beijing, China; 3 Biomedical Imaging Center, Rensselaer Polytechnic Institute, Troy, New York, United States of America; University of Zurich, Switzerland

## Abstract

The study of light propagation in turbid media has attracted extensive attention in the field of biomedical optical molecular imaging. In this paper, we present a software platform for the simulation of light propagation in turbid media named the “Molecular Optical Simulation Environment (MOSE)”. Based on the gold standard of the Monte Carlo method, MOSE simulates light propagation both in tissues with complicated structures and through free-space. In particular, MOSE synthesizes realistic data for bioluminescence tomography (BLT), fluorescence molecular tomography (FMT), and diffuse optical tomography (DOT). The user-friendly interface and powerful visualization tools facilitate data analysis and system evaluation. As a major measure for resource sharing and reproducible research, MOSE aims to provide freeware for research and educational institutions, which can be downloaded at http://www.mosetm.net.

## Introduction

The optical molecular imaging technique, being able to reveal molecular and cellular activities in a small animal *in vivo*, has grown into an important tool in biomedical research [1]–[3]. Typical optical molecular imaging modalities include bioluminescence imaging (BLI) and fluorescence molecular imaging (FMI). These technologies allow the investigation of specific molecular and cellular events, such as cell migration, signal transduction, proliferation and apoptosis [4]. The 3D reconstruction of the distribution of bioluminescent or fluorescent molecules, known as the inverse problem of bioluminescence tomography (BLT) [5] or fluorescence molecular tomography (FMT) [6], provides biologists with a more accurate quantitative analysis of an *in vivo* study of metabolism. Diffuse optical tomography (DOT) [7] is another kind of optical molecular imaging technique. As an optical tomography approach, DOT uses absorption and scattering as the source of contrast. Its fluorescence counterpart, FMT, exploits the fluorescence emission from probes or proteins [8]. According to the modulation modality of the light source, the optical molecular imaging technique operates in three modes: continuous wave (CW), time domain (TD), and frequency domain (FD), corresponding with a constant intensity light source, short duration laser pulse, and a low frequency modulated light source respectively [9]. Both DOT and FMT can operate with TD, FD and CW modes. BLT can only work with the CW mode due to the specific property of luciferase. The devices and procedures of the three optical molecular imaging techniques are different.

The study of light propagation in turbid media plays an important role in optical molecular imaging [10] and has been extensively investigated [11], [12]. Generally, methods for solving the problem of light propagation can be evaluated with real imaging experiments. Due to the limitations of availability and expense of the real imaging experiment, researchers seek for low-cost computer simulation approaches. By simulating light propagation in turbid media with a computer, the resultant accurate and reliable results can be obtained and used as standards for the newly developed method. This not only reduces research costs, but also provides more flexibility and higher efficiency. Computer simulation is a powerful tool for researchers in the study of light propagation in optical molecular imaging.

It is well known that the radiative transfer equation (RTE) is highly accurate for the description of light propagation in turbid media. However, it is difficult to solve numerically even though there are various approximate models [13]–[15]. The Monte Carlo (MC) method can solve RTE with desired accuracy and is employed as the gold standard for light propagation [16]. Several light propagation simulation tools based on the MC method have been developed [17]–[22]. A Monte Carlo model that describes steady-state light transport in multi-layered tissues (MCML) was developed by Wang *et al* [17], where a narrow light beam perpendicularly illuminated the tissues. In the model, each layer tissue was considered as an infinitely wide flat area with different photonic parameters. Although widely used, MCML can only deal with multi-layered tissues in two dimensions. Boas *et al.* set up a three dimensional Monte Carlo code (tMCimg) [18] which calculated the migration of light through 3D highly scattering media with arbitrary complex media represented by voxelated space. However, the density of the voxel became extremely large when the modeling regions were with curved boundaries. In order to deal with the regions had curved boundaries, the triangle-based or tetrahedron-based Monte Carlo program was developed [19], [20]. However, these programs were specifically developed for scientific intentions without a graphic user interface for visualization. Some general-purpose Monte Carlo radiation transport softwares such as MCNP [21], GEANT4 [22] *etc* were also published, which were used for neutron, photon, electron, or coupled neutron/photon/electron transport. Unfortunately, they were overly complex for most common users to use.

In cooperation with Xidian University, Institute of Automation at the Chinese Academy of Sciences, China and Rensselaer Polytechnic Institute, USA, we present a simulation tool called the Molecular Optical Simulation Environment (MOSE) for light propagation and data analysis. It can describe the entire transport procedure of light, including the generation from different light sources, the propagation through turbid media and the detection event caused by the charge coupled device (CCD) at multiple angles. It supports the simulation of BLT in a CW mode as well as DOT and FMT in TD, FD, and CW modes. It has a graphic user interface with real-time visualization of the simulation environment and the results, which makes it easy and convenient for users. In addition to the main function of light transport simulation, it also supports other functions of imaging processing, mesh simplification, and surface reconstruction.

## Methods

**Figure 1:**
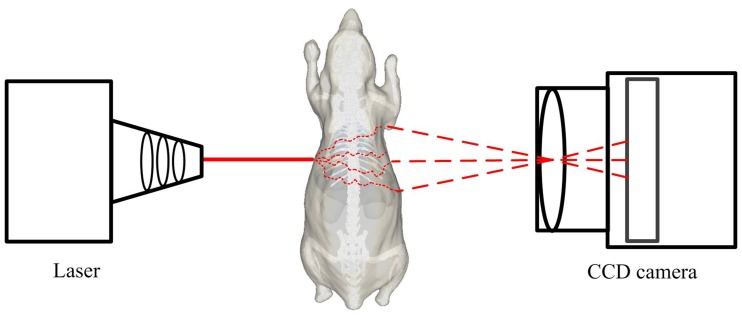
Process of light transport in turbid media and outer space. The laser is employed as the light source in DOT and FMT systems. It is not necessary when the system is a BLT system. The path of the photons is twisted because the absorption and scattering events happen when the light transports in turbid media. The path of photons is straight when the light is transported in outer space.

The entire light transport in the simulation environment can be divided into two parts (Figure 1). One is photon transport in the inner space of the tissues, which is described by the Monte Carlo method. The other is light transmission from the tissue surface to the charge coupled device (CCD), which can be modeled by the radioactive Lambertian source theory [23], [24] and pinhole imaging.

### Monte Carlo method

The MC method provides approximate solutions to a variety of mathematical problems by performing statistical sampling experiments. The statistical sampling utilizes sequences of random numbers to perform the experiments. The process of the MC method involves performing many simulations using random numbers and probability to get an approximate solution to the problem.

In the MC method, light is dispersed into a group of photon packets. The MC model of light propagation describes the local rules of photon propagation that are expressed as probability distributions of the photon's behavior, such as the step size of the movement between sites of photon-tissue interaction, and the angles of deflection in a photon's trajectory when a scattering event occurs. The simulation can record multiple physical quantities simultaneously. However, the method is statistical in nature and relies on calculating light propagation with a large number of photons. As a result, this method requires a large amount of computation time.

**Figure 2:**
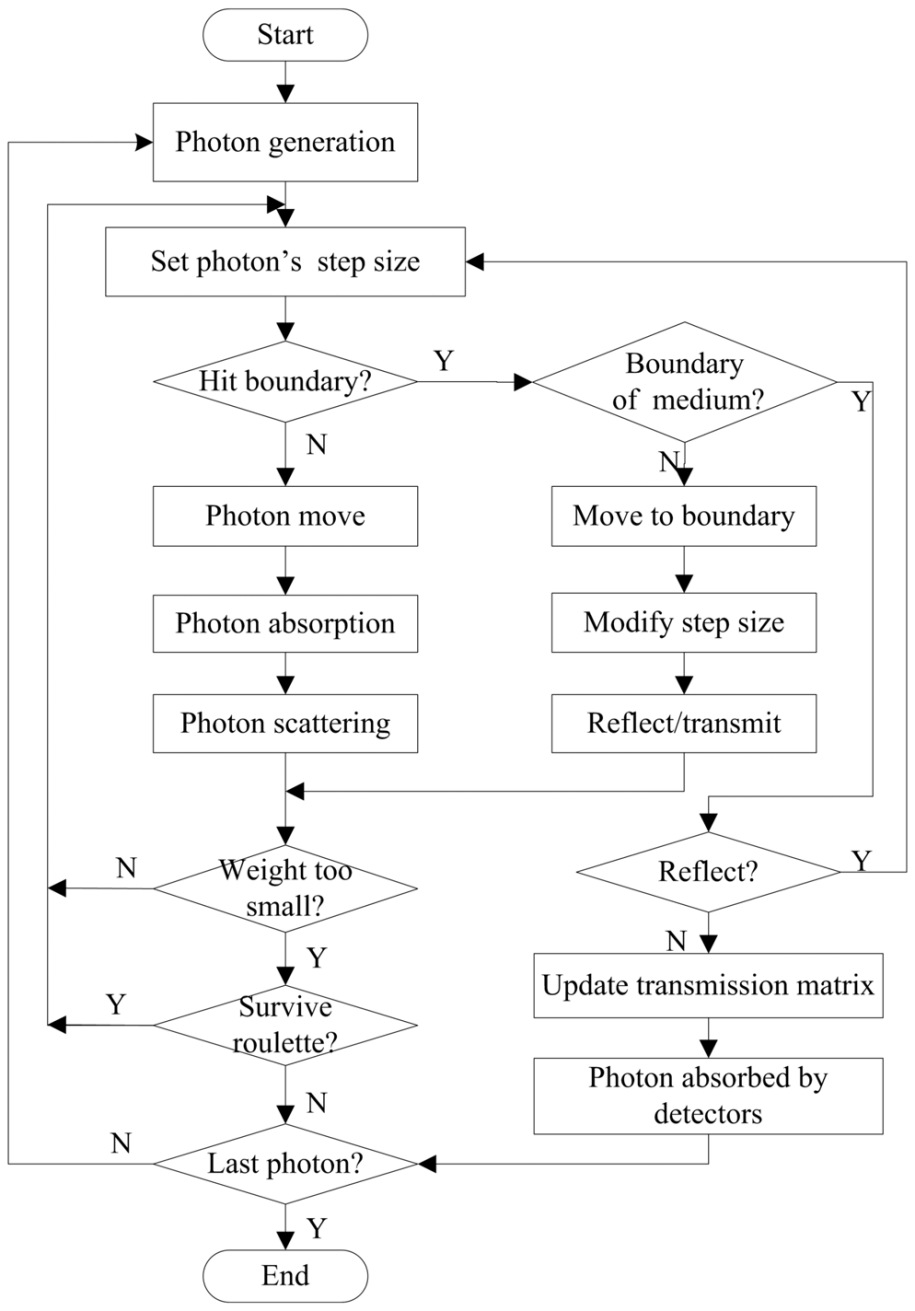
Flow chart of the Monte Carlo simulation of light propagation in turbid media.

The implementation of the MC method in MOSE for simulation of photon propagation in turbid media is similar to that used in MCML [17]. The major difference is that triangular meshes are used to model the complicated tissues in MOSE [19]. The transmittance in MOSE is recorded in each triangular mesh. The flowchart for the simulation of photon propagation in turbid media in MOSE is shown in Figure 2.

When a new photon packet is launched from the light source, it is assigned an initial weight, which is determined by dividing the total power of the light source by the number of photons. The initial position of the photon is determined by a random sampling of the region of the light source. The initial direction of the photon is determined by an azimuthal angle which is uniformly distributed over 0 to 2π and a deflection angle which is distributed from 0 to π. When the photon travels in the tissues, the step size of the photon can be calculated by the following function:
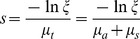
(1)where *μ_a_* is the absorption coefficient, *μ_s_* is the scattering coefficient, and *ξ* is a random number distributed uniformly from 0 to 1.

For each step, the photon will be absorbed, scattered, and reflected by the tissue boundary or transmitted out of the tissue boundary. Then, the step will be repeated until the photon is totally absorbed or transmitted out of the tissue boundary. The deflection of the photon in each step is determined by the Henyey-Greenstein phase function [25]. The cosine value of the deflection angle can be calculated as:
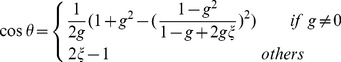
(2) where *ξ* is a random number normally distributed in the interval [0, 1]; *g* is the anisotropy factor.

The photon may be reflected when it travels through a boundary between two tissues with different refractive indices. Whether the photon will be reflected or transmitted is determined by the critical angle *θ_c_* and the internal reflectance *R*(*θ_i_*). The critical angle *θ_c_* is calculated as:
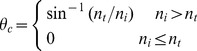
(3)where *n_i_* and *n_t_* are the relative refractive indices of the medium from which the photon is incident and transmitted.

The internal reflectance *R*(*θ_i_*) is calculated according to the Fresnel reflection coefficient:
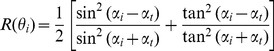
(4) where *α_i_* and *α_t_* are the incidence angle and transmission angle respectively. A uniform unit random number *ξ* is compared with *R*(*θ_i_*). If *ξ*≤*R*(*θ_i_*), the photon will be reflected totally, otherwise it will be transmitted totally. Once the photon is transmitted, the boundary will be checked. If the boundary is the outermost boundary, the photon will be terminated and the residual power of the photon will be recorded in a transmission matrix. If the boundary is the internal boundary, the photon will continue propagating with updated direction and step.

There is another way to terminate the photon's propagation. The photon can be absorbed completely by the tissues. When its weight drops below the predefined threshold, the Russian roulette technique is used to determine whether the photon will survive: 
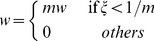
(5)where *ξ* is a random variable uniformly distributed over the interval [0, 1]; *m* is a constant number defined by users. If the photon survives, the power of the photon will be set to *mw* and the propagation will continue. Otherwise, the photon will be terminated and a new photon will be set up.

### Light propagation in free space

When the photon transmits out of the tissue surface, it will be transmitted in free space where no absorption and scattering effects exist. A free space photon propagation model was proposed by Ripoll [26]. Chen *et al.* [27] proposed a model based on the hybrid radiosity-radiance theorem,which combines Lambert's cosine law and the radiance theorem. The results obtained by Chen's model are more similar to the physical measurements compared with Ripoll's model. The model of light transport in free space used in MOSE is similar to Chen's model. The distribution of the flux density on the detector plane is determined by the flux density of the tissue surface and the positional relationship between the detector and the tissue. The model of light transport in free space of MOSE includes position mapping which is based on the pinhole imaging principle and energy mapping which is based on the Lambertian source theory.

**Figure 3:**
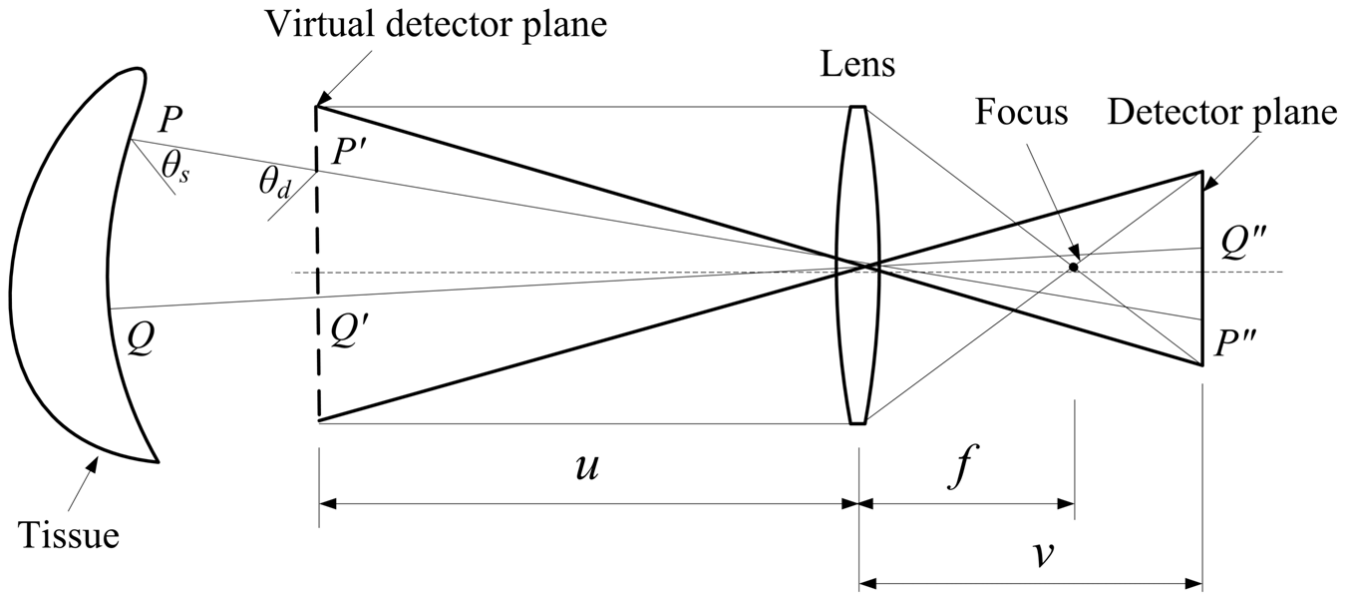
Light transport in free space.

First, we need to determine the specific positional relationship between the media surface and CCD camera. As shown in Figure 3, *P* and *Q* are the points on the tissue surface; *u*, *v* and *f* are the object distance, image distance, and the focus of the lens respectively. The line *PP′* which travels through the center of the lens intersects the virtual detector plane at *P′* and the detector plane at *P″*. According to the pinhole imaging principle, the distance between *P′* and the axis is proportional to that of *P″* and the axis. We only have to determine the position of the intersection point *P* on the tissue surface.

The flux density of point *P″* is equal to that of point *P′* according to the positional relationship. The flux density of a differential receiving area *dA* at point *P′* can be calculated with Lambert's cosine law.
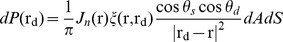
(6)where r and r_d_ are the centers of *P* and *P'*; *θ_s_* is the angle between the differential surface normal and the directional vector from r to r_d_; *θ_d_* is the angle between the differential detector normal and the directional vector from r to r_d_, and *dS* and *dA* are the area of the differential surface and receiving unit respectively. *ξ*(r, r_d_) is a visibility factor that discards the surface points invisible from the receiving plane. The detector plane is constructed by several pixels and the flux density of each pixel is calculated according to the rules above.

### Implementation, Usage, and Requirements

**Figure 4:**
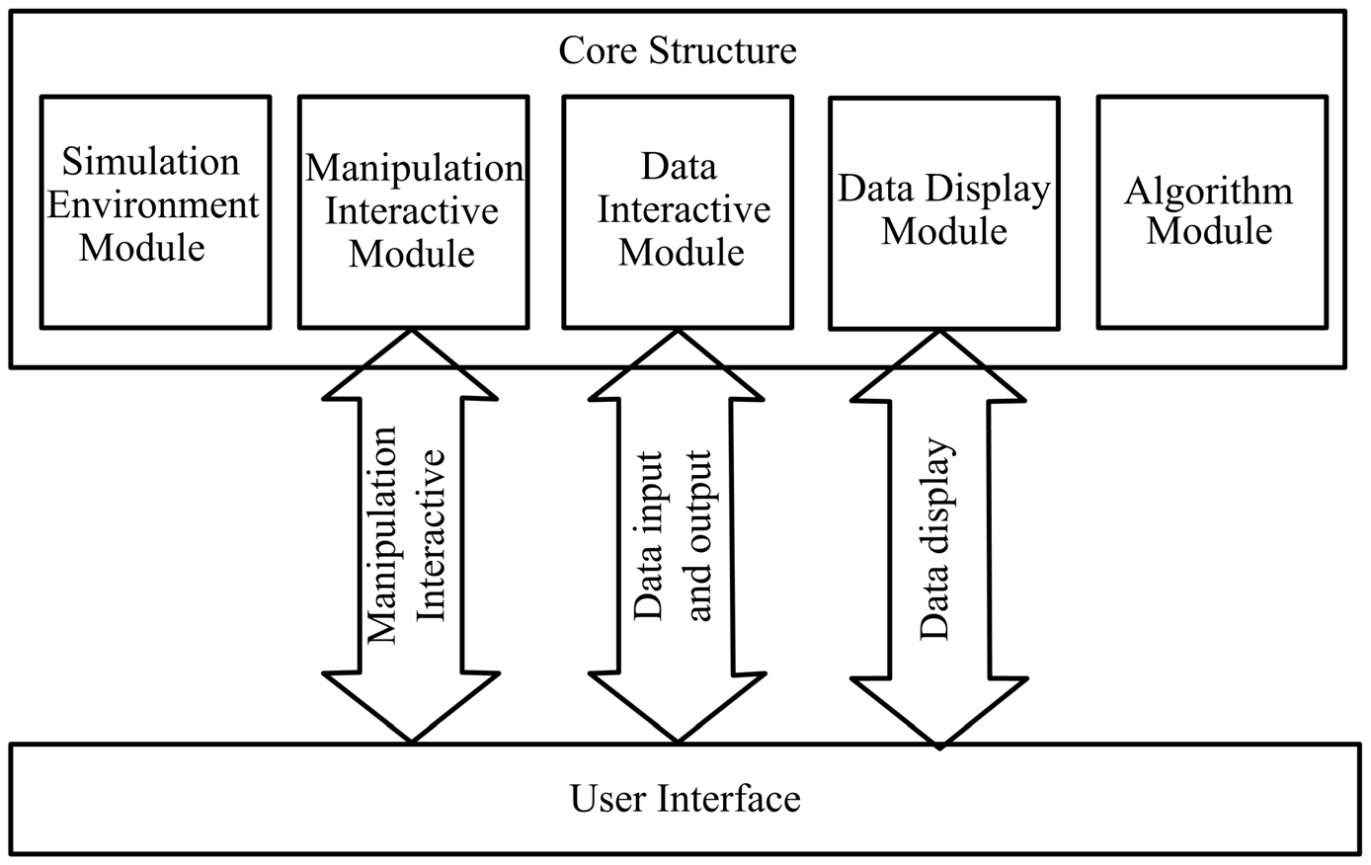
Framework of MOSE. The main functions of MOSE are implemented by the core structure and the user interface. On the basis of the core structure, simulation data can be processed and displayed easily using the user interface.

The framework of MOSE can be divided into two parts, the core structure and the graphic user interface (GUI). The core structure of MOSE was developed by standard C++ and packaged as a Dynamic Linkable Library (DLL). The core structure can be divided into five modules (see Figure 4), including the simulation environment module, algorithm module, manipulation interactive module, data interactive module and data display module. Since the core structure of MOSE is packaged as a DLL, it is easy to develop GUI in different platforms. In the current version of MOSE, GUI was developed by using the Microsoft Foundation Classes (MFC) which are supported by the Microsoft cooperation. The phantom data and simulation results data are rendered graphically by using the Open Graphics Library (OpenGL).

**Figure 5:**
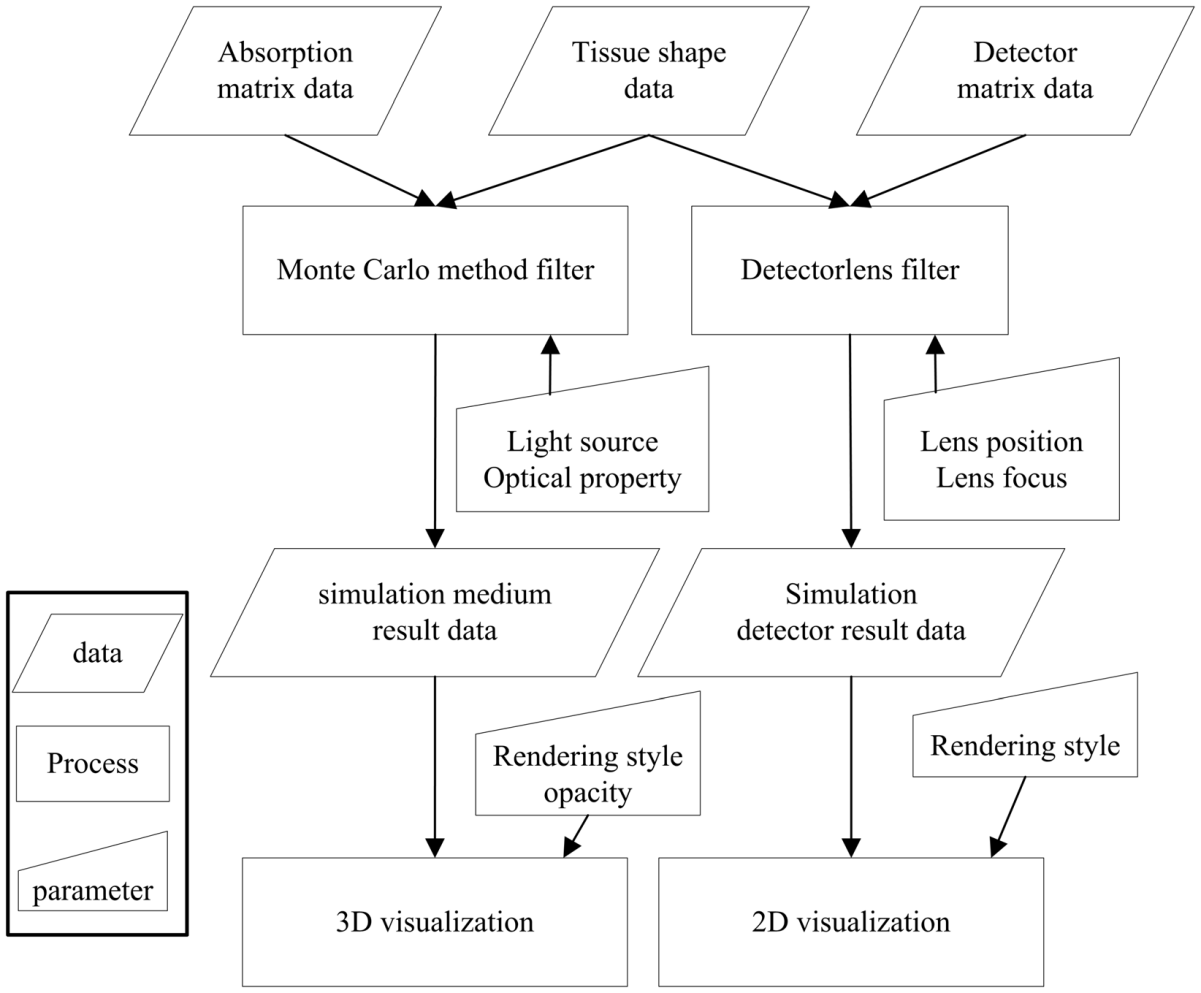
Simulation module and data flow of MOSE. The medium result data contains the absorption and transmittance data which can be rendered for 3D visualization. The detector result data can be rendered for 2D visualization.

**Figure 6:**
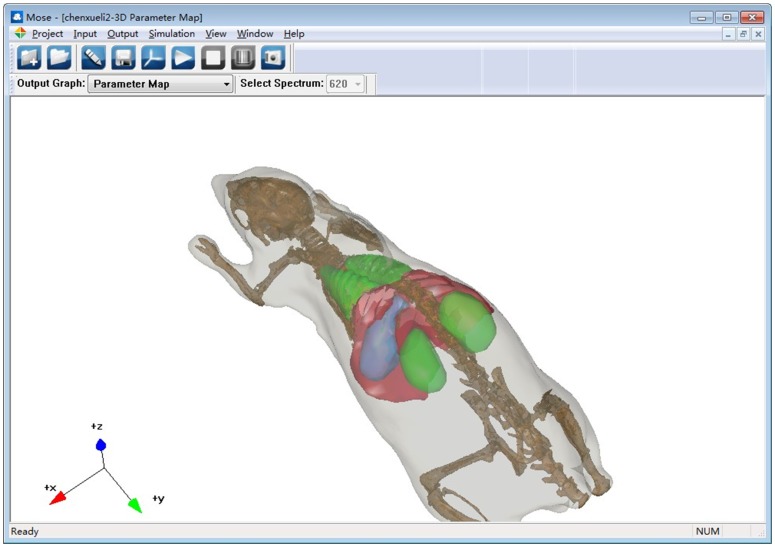
Main interface of MOSE.

**Figure 7:**
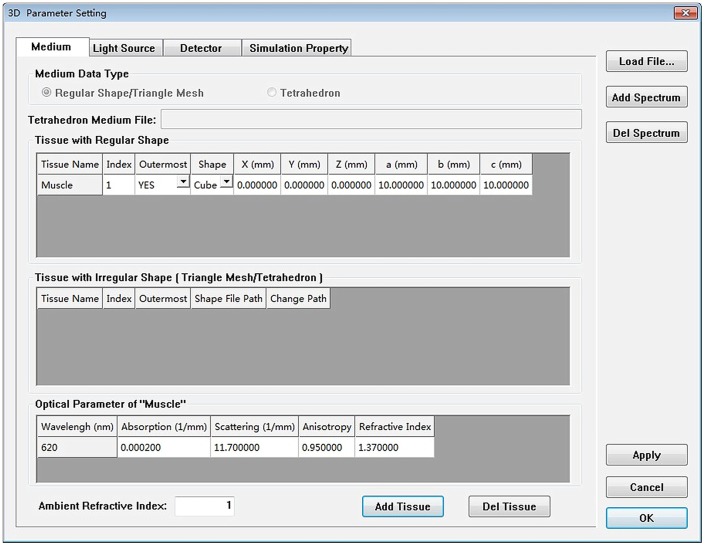
Parameter inputting panel of MOSE.

The simulation module mainly contains the process of the Monte Carlo simulation and the process of light propagation in free space. Figure 5 shows the data flow and the typical simulation module of the application. The main graphic user interface of MOSE is shown in Figure 6. To set up a simulation project of optical molecular imaging, the parameter of the simulation is needed, including the information on tissues, light source, detector and simulation property. There are two ways to input the simulation parameters: reading from the simulation parameter file or inputting the simulation parameter manually in the parameter panel box (see Figure 7). Loading a parameter file from the disk is convenient for users to use. However, the parameter file has a specified form and should be prepared manually first. The parameter panel box includes four parts: the medium panel, light source panel, detector panel, and simulation property panel. Users should add medium information first. Then, optical parameters of the tissues and light source information should be added. The detector is optional for a light transmission simulation, which can be chosen by users. When all the simulation parameter information is finished, a temporary parameter file is written on the disk. Then, users can start the simulation manually. When the simulation is over, the simulation result data can be written on the disk and viewed by users.

The software can run on most personal computers with Windows 7 or Windows XP operation systems, with at least 512 MB of memory and 250 MB of free disk space. The software uses a temporary path-setting file (path.ini) to specify the disk path of the simulation project.

## Results

In this section, three simulations of light propagation in turbid media were conducted to illustrate the functions of MOSE. The photon number of the light source was set to be ten million for all of the simulations.

### Example 1: Simulation of BLT in continuous wave mode

**Figure 8:**
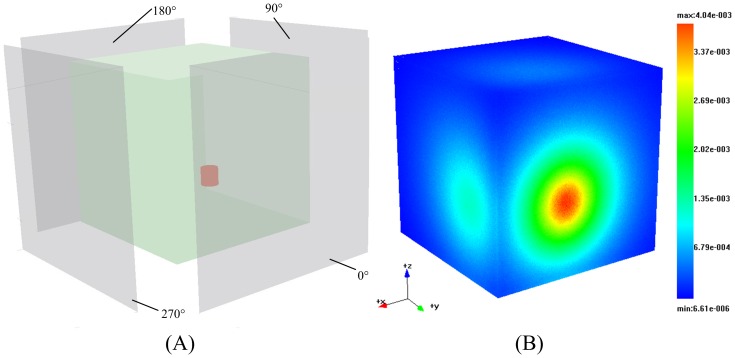
Simulation of BLT. (A) Phantom structure of the simulation in MOSE. The detectors around the phantom are located at angles 0°, 90°, 180°, and 270° respectively. (B) Transmittance results of the simulation in MOSE. The region of the red line is sampled for the quantitative comparison of the simulation for MOSE and the real experiment.

The first simulation sample was conducted to simulate the BLT simulation in continuous wave mode by using MOSE. As shown in Figure 8, a cube-shaped uniform phantom was utilized for the simulation. The size of the phantom is 10 mm×10 mm×10 mm. The light source of the simulation was located near the center of the phantom with a 3 mm distance to the surface. Four CCD detectors were located around the phantom with an angular interval of 90 degrees. In order to validate the accuracy of the Monte Carlo simulation of light propagation, we conducted a real experiment by using a highly sensitive charge-coupled device (CCD) camera. The phantom of the real experiment, which is made from polyethylene, has the same size and shape as in the simulation. The optical parameters of the phantom, described by the absorption coefficient (*µ_a_*), scattering coefficient (*µ_s_*), anisotropy coefficient (*g*) and refractive index (*n*) of the phantom, are 0.0002 mm^−1^, 11.7 mm^−1^, 0.95 and 1.37 respectively at the wavelength of 620 nm, which were measured by diffuse optical tomography. The light source of the real experiment is the fluorescence with a peak spectrum around a 600 nm wavelength. A 40 nm band-pass filter centered at 620 nm coupled with the lens of the camera was used during image acquisition.

We used the Parameter inputting panel of MOSE to set up the simulation. The medium information of the simulation is shown in Figure 7. The photons of the light source are uniformly distributed. Each surface of the phantom is separated as a 300×300 matrix for transmittance recording. After finishing all of the simulation parameter information, the temporary parameter file of the simulation was written onto the disk. The parameter file contains all of the simulation information, including the simulation property, spectrum, light source, medium and detector information.

**Figure 9:**
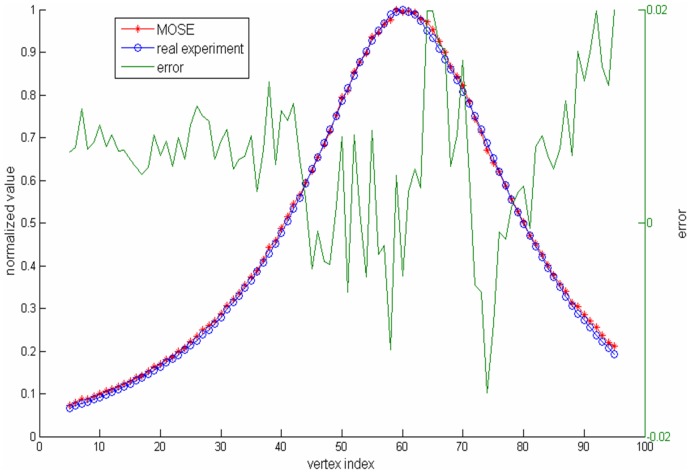
Normalized flux density around the red line area between the simulation results and the real experiment.

**Figure 10:**
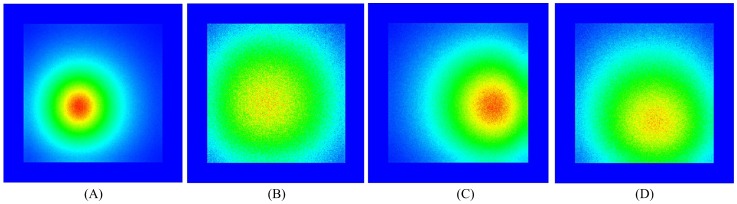
Flux density of the detector results of the simulation at different angles.

The transmittance results of the simulation are shown in the right area of Figure 8. The results are compared with those of the real experiment. Figure 9 shows the normalized flux density around the red line area between the simulation results and the real experiment, where we can see that the real experiment and the simulation results have good agreement. The normalized root mean square error (NRMSE) was utilized to estimate the discrepancy between the two results.
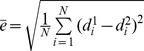
(7)where 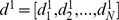, 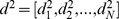, and *N* is the dimension of the resulting data. The NRMSE between the two results was 0.009. The flux densities of the detectors of the simulation at four different angles are shown in Figure 10.

### Example 2: Simulation of DOT in continuous wave mode

**Figure 11:**
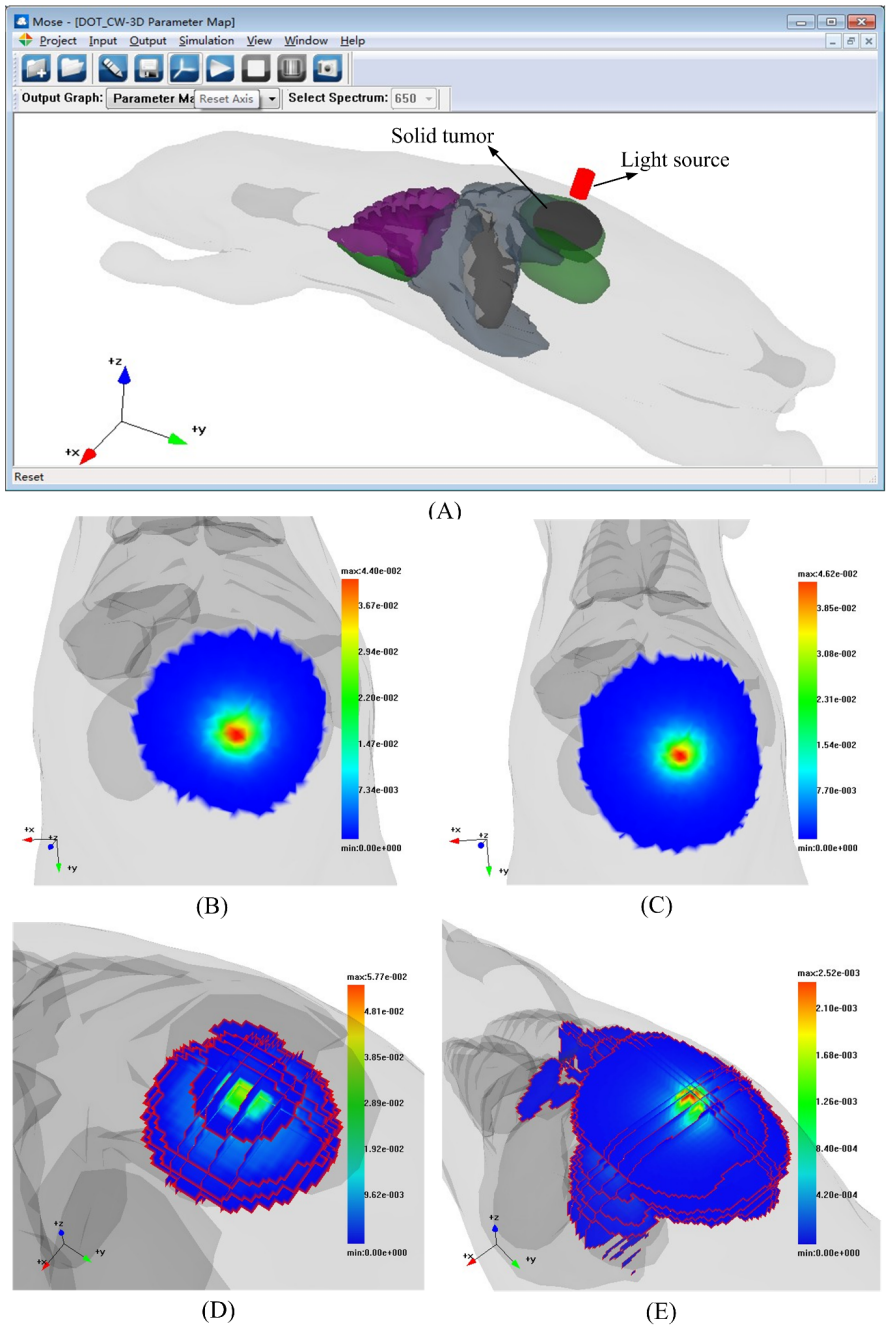
Simulation of DOT. (A) Phantom structure of DOT simulation. The light source colored in red is located near the back of the digital mouse. The solid tumor colored in dark gray is located on the inside of the digital mouse. (B) Reflectance results of the DOT simulation with the solid tumor in the digital mouse. (C) Reflectance results of the DOT simulation in the normal tissues. (D) Absorption results of the DOT simulation in solid tumor of the digital mouse. (E) Absorption results of the DOT simulation in the normal tissues.

The second simulation was conducted to illustrate the function of the DOT simulation in continuous wave mode by using MOSE. DOT has been used for detection and diagnosis of breast cancer [28]. The difference between solid tumors and normal tissues is that solid tumors show concurrent higher absorption and scattering related to the normal tissues. In this simulation, a digital mouse depicted by the triangular meshes with six normal tissues was utilized as a phantom. An assumed solid tumor depicted by the shape of an ellipsoid was located on the back of the digital mouse. The light source of the simulation is located outside of the digital mouse and it illuminates the back of the mouse perpendicularly (see Figure 11A). In order to solve the inverse problem of DOT, the forward problem for DOT should be solved first and the assumed optical parameters should be given first. The assumed optical parameters of the digital mouse under the light source at a spectrum of 670 nm are shown in Table 1. The reflectance of the simulation result with the solid tumor is shown in Figure 11B. The reflectance of the corresponding simulation results with normal tissues is shown in Figure 11C. Figure 11D shows part of the internal absorption results of the simulation with a solid tumor, which are depicted by a multilayer. Figure 11E shows part of the internal absorption results of the corresponding simulation with normal tissues. The resolution of the region of interest was 0.5 mm×0.5 mm×0.5 mm.

**Table 1:** Optical properties of the phantom of DOT simulation.

Tissue	*µ_a_*(mm^−1^)	*µ_s_*(mm^−1^)	*g*	*n*
Surface	0.004	20.13	0.94	1.37
Lung	0.196	36.23	0.94	1.37
Kidney	0.066	16.09	0.86	1.37
Heart	0.059	6.42	0.85	1.37
Stomach	0.011	17.96	0.92	1.37
Liver	0.035	6.78	0.9	1.37
Solid tumor	0.55	29.5	0.9	1.37

### Example 3: Simulation of FMT in the time domain

**Figure 12:**
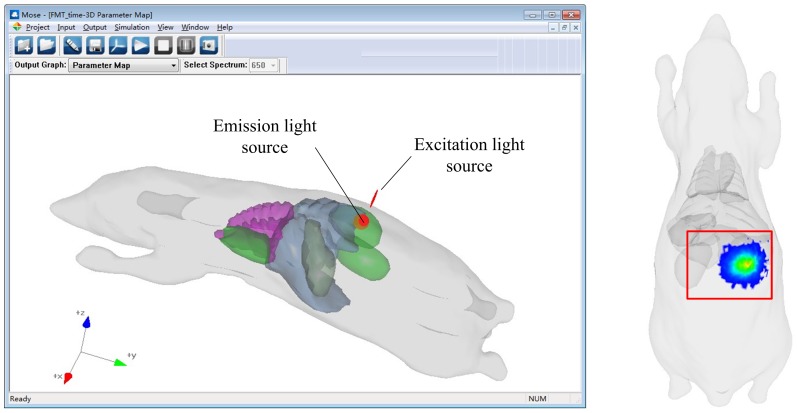
Phantom structure of the simulation of fluorescence molecular tomography and the transmittance results.

**Figure 13:**
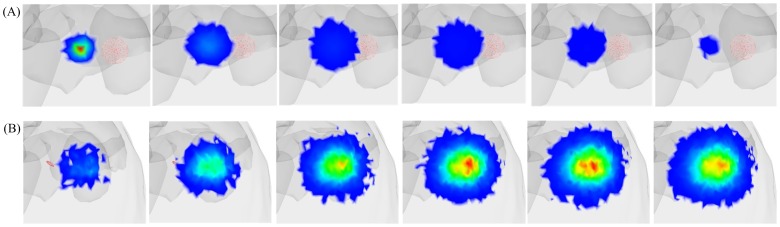
Transmittance results for the simulation of FMT. (A) Transmittance results of the light source at the excitation wavelength from 10 ps to 60 ps. (B) Transmittance results of the light source at the emission wavelength from 50 ps to 100 ps

The third simulation was conducted to illustrate the function of the FMT simulation in the time domain. FMT can quantify and localize fluorophore distribution in small animals [29]. Time domain fluorescence molecular imaging specifically allows non-invasively and quantitative tracking of specific molecular events *in vivo* [30]. This technology demonstrated that it had the capability to detect differences in the probe delivery route in small animals *in vivo* [31]. The phantom is the same as that used in Example 2. The optical parameters of the digital mouse under the excitation light source at a spectrum of 620 nm and the emission light source at a spectrum of 690 nm are shown in Table 2. The emission light source is located in the right kidney and the excitation light source is located on the outside of the mouse surface (see Figure 12). The excitation light source illuminates the back of the digital mouse perpendicularly. When the excitation photon travels in the area of the emission light source, the probability that the photon of the emission light source will be excited is determined by the quantum efficiency of the emission light source and a random number. Once the photon of the emission light source is excited by the excitation photon, the excitation photon will be terminated. The quantum efficiency of the emission light source of the simulation is set to be 0.6. The simulation time is from 0 ps to 100 ps and the interval of the time domain is 10 ps. The transmittance results of the light source at the excitation wavelength from 10 ps to 60 ps are shown in Figure 13A. The transmittance results of the light source at the emission wavelength from 50 ps to 100 ps are shown in Figure 13B.

**Table 2:** Properties of the optical parameters under the spectrum of the excitation source (620 nm) and the emission source(690 nm).

Tissue	620 nm				690 nm			
	*µ_a_*(mm^−1^)	*µ_s_*(mm^−1^)	*g*	*n*	*µ_a_*(mm^−1^)	*µ_s_*(mm^−1^)	*g*	*n*
Surface	0.0088	20.97	0.94	1.37	0.003	19.82	0.94	1.37
heart	0.138	7.18	0.85	1.37	0.04367	6.16	0.85	1.37
stomach	0.026	19.36	0.92	1.37	0.0086	17.46	0.92	1.37
lung	0.46	37.75	0.94	1.37	0.145	35.67	0.94	1.37
liver	0.829	7.35	0.9	1.37	0.261	6.57	0.9	1.37
kidney	0.155	18.09	0.86	1.37	0.05	15.39	0.86	1.37

## Discussion

In this paper, we developed an optical molecular imaging simulation platform called the Molecular Optical Simulation Environment (MOSE) which can simulate light transport both in tissues and free space. The application examples showed that MOSE has the capability to simulate various modes of light propagation. Although the Monte Carlo method has been considered as the gold standard for light propagation, the results of the first example showed that there still was a little difference between the simulation of BLT by using MOSE and the real experiment. This might be caused by the following reasons. First, although the measurement was obtained with a 40 nm band-pass filter, the spectrum of the light source is still very wide, which may have caused the optical properties of the phantom at a spectrum of 620 nm to not be suitable for the simulation. Second, the non-calibration of the CCD camera might also have caused the difference. Images obtained by the CCD camera can be influenced by both the bright and dark fields. Because of these factors, the simulation results of MOSE are a little steep than that of the real experiment (see Figure 9).

The results of DOT simulation in Example 2 show that the solid tumor and normal tissues can cause different reflectance results because of their different optical properties. The reflectance results of DOT simulation with the solid tumor are lower and smaller than that of the normal tissues. However, to quantitatively analyze the molecular events *in vivo*, the inverse problems of DOT should be solved more precisely. The study of the forward problems of DOT and its fluorescence counterpart, FMT, is the precondition of the study of the inverse problems. MOSE is capable of solving the forward problems of DOT and FMT based on the MC method. To solve the inverse problem of FMT, the Jacobian for FMT should be prepared first, which can be calculated by the absorption of the excitation light and emission light [32]. The results obtained in example 3 contain the time-resolved absorption and transmittance of the excitation light and the emission light, which makes MOSE a powerful tool for the study of FMT. The results of example 2 also show the capability of MOSE in a study of DOT.

There is also some other Monte Carlo software developed for simulation of light propagation. TIMOS is an MC simulator based on tetrahedral meshes [20]. It has many advantages over the model based on triangular meshes which is currently used in MOSE. The time spent on searching the triangle for the ray-triangle intersection step in MC simulation has been reduced largely. Each of the tetrahedrons can be set with the typical optical properties. However, the tetrahedrons should be constructed first. With the development of network technology, the cloud-computing system provides users with high computing efficiency and convenient services. Doronin *et*. *al*. developed an online MC computational tool based on peer-to-peer MC simulation [33]. Users can access this MC simulation tool by using the online user interface. The web server hosts the online user interface and accepts MC simulation requests from users. The web-based MC platform may attract much more attention in the future.

On the other hand, we acknowledge that the Monte Carlo method based simulation suffers from low computational efficiency. To remedy this, we have implemented the parallel acceleration of the Monte Carlo simulation in MOSE using a multi-core CPU with open multi-processing (OpenMP), but it still takes a long time when it comes to a large-scaled simulation of light propagation in tissues. In the past years, the graphics processing units (GPU) based acceleration technique has attracted intense attention because of GPU's much higher raw processing power as well as a relatively low cost. Based on the Compute Unified Device Architecture (CUDA) platform developed by NVIDIA [34], we have developed a GPU-based Monte Carlo simulation method for light propagation in turbid media [19]. The GPU acceleration version of MOSE is under development.

In general, we have developed a simulation platform for light propagation in turbid media. MOSE is able to simulate light propagation in both turbid media with complicated structures and free space based on the Monte Carlo method and Lambertian source theory. The graphic user interface of MOSE provides a friendly interface to input the simulation parameters of a project and a 3D visualization of the simulation results. The simulation of BLT in CW mode and the simulation of DOT as well as FMT in the CW, TD and FD modes can be easily conducted by MOSE, which makes MOSE a very useful tool for studying light propagation.
